# A Systematic Review of the Relationship between Serum Vitamin D Levels and Caries in the Permanent Teeth of Children and Adolescents

**DOI:** 10.3390/dj12040117

**Published:** 2024-04-22

**Authors:** Roxana Buzatu, Magda Mihaela Luca, Bogdan Andrei Bumbu

**Affiliations:** 1 Department of Dental Aesthetics, Faculty of Dental Medicine, “Victor Babes” University of Medicine and Pharmacy Timisoara, Revolutiei Boulevard 9, 300041 Timisoara, Romania; roxana.buzatu@umft.ro; 2 Department of Pediatric Dentistry, Faculty of Dental Medicine, “Victor Babes” University of Medicine and Pharmacy Timisoara, Eftimie Murgu Square 2, 300041 Timisoara, Romania; 3 Department of Dental Medicine, Faculty of Medicine and Pharmacy, University of Oradea, 410073 Oradea, Romania; bogdanbumbu@uoradea.ro

**Keywords:** vitamin D, dental caries, permanent dentition, child, adolescent, stomatology

## Abstract

This systematic review critically evaluates the association between serum Vitamin D levels and dental caries incidence in the permanent teeth of children and adolescents. The search strategy comprised three databases (PubMed, Scopus, Embase), up to November 2023, targeting studies on the correlation between Vitamin D and dental caries in permanent dentition. The eligibility criteria focused on observational studies involving children and adolescents aged 12 to 19 years with permanent dentition. The screening process, guided by the PRISMA guidelines and the Newcastle–Ottawa Scale for quality assessment, resulted in the inclusion of eight studies conducted across various global regions from 2013 to 2023. The analysis revealed that Vitamin D insufficiency and deficiency were prevalent among the study populations, ranging from 17.3% to 69.4%. Specifically, children and adolescents with Vitamin D insufficiency (<50 nmol/L) were found to have significantly higher odds of developing caries, with odds ratios (ORs) ranging from 1.13 to 2.57. Conversely, two studies indicated a protective effect of higher Vitamin D levels, with an OR of 0.80 and 0.59, respectively, for caries among children and adolescents with serum levels ≥ 50 nmol/L, suggesting an inverse relationship between Vitamin D status and caries risk. The results indicate both the protective role of adequate serum levels of Vitamin D above 20 ng/mL and the increased risk associated with insufficient levels below this threshold. However, the variations in study quality, methodologies and geographic settings underscore the challenges in drawing universal conclusions. Despite these limitations, our review suggests that improving Vitamin D status could be a beneficial component of preventive strategies against dental caries in children and adolescents, warranting further research to clarify the clinical significance of our findings.

## 1. Introduction

The relationship between micronutrient status and oral health outcomes has been a subject of considerable scientific inquiry, with Vitamin D emerging as a micronutrient of significant interest [1,2,3]. Vitamin D plays an essential role in bone metabolism and mineralization, processes that are important for the development and maintenance of healthy teeth [4,5]. It has been hypothesized that adequate serum levels of Vitamin D may be protective against dental caries, particularly in the permanent teeth of children and adolescents, who are at a critical stage of dental development [6,7].

Dental caries, a multifactorial disease, remains one of the most common chronic diseases among children and adolescents worldwide [8]. It results from the demineralization of tooth enamel due to the acidic by-products of the bacterial metabolism of dietary sugars [9]. The prevention and management of dental caries are of paramount importance in pediatric dentistry given its impact on oral health, general well-being, and associated healthcare costs [10,11]. Emerging evidence suggests that Vitamin D, through its role in calcium and phosphate metabolism, may influence the resistance of teeth to cariogenic bacteria and the demineralization process [12,13].

The protective role of Vitamin D against dental caries may also be attributed to its influence on the overall immune system and the modulation of inflammatory responses [14], modulating the adaptive immune response, which could contribute to the prevention of infections in the oral cavity, including those that lead to caries development [15]. Additionally, in the context of public health, dental caries can be present in as many as 85% of 12-year-olds, with expenditures up to 10% of the public health budgets for developed countries [16,17]. Therefore, understanding the potential impact of Vitamin D on dental caries could inform preventive strategies and nutritional recommendations aimed at reducing the incidence of this condition among children and adolescents [16].

Despite the biological plausibility of Vitamin D’s protective role against dental caries, empirical evidence remains mixed, with studies reporting varying degrees of association. Moreover, permanent teeth, which begin to appear and reach stability during adolescence, present a unique opportunity to study the impact of Vitamin D on dental health, free from the confounding variables present in younger, deciduous teeth-bearing individuals [18]. This distinction is essential, as it allows for an investigation into a phase of dental development where the effects of Vitamin D, correlated with the backdrop of significant dietary and hormonal changes pertinent to this age group, on dental caries can be examined with greater clarity and specificity. Targeting this demographic ensures that the study’s findings are relevant and applicable to those with a fully emerged permanent dentition. 

Therefore, this systematic review aims to critically evaluate the existing literature on serum Vitamin D levels and their correlation with dental caries incidence in permanent teeth among children and adolescents. The primary endpoint was to establish whether serum Vitamin D deficiency can be considered a risk factor for caries development in the permanent teeth of children and adolescents. The secondary endpoint was to determine other influencing factors of caries development in this population.

## 2. Materials and Methods

### 2.1. Protocol and Registration

This study employed a search strategy that was used across all three electronic databases (PubMed, Scopus, and Embase). The search aimed to encompass studies published up to November 2023, to include the latest available studies on this topic. In this study, the PICO framework centers on adolescents aged 12 to 19 years with permanent dentition (population) to explore the impact of serum Vitamin D levels on the incidence of dental caries in these individuals (intervention). By comparing those with varying levels of Vitamin D—sufficient versus insufficient or deficient (comparator)—this study aims to understand the relationship between Vitamin D status and dental caries occurrence in permanent teeth (outcome).

The search strategy was developed to include a set of keywords and phrases based on the study’s objectives, focusing on the correlation between Vitamin D levels and dental caries, including the following: “Vitamin D”, “serum 25(OH)D”, “calcidiol”, “dental caries”, “tooth decay”, “permanent teeth”, “primary teeth”, “children”, “adolescents”, “pediatric population”, “dental health”, “oral hygiene”, “micronutrients”, “nutritional status and dental health”, “Vitamin D deficiency and dental caries”, “pediatric dentistry”, “oral health”, “caries prevention”, “nutritional influences on dental health”, “calcium metabolism”, “phosphorus metabolism”, “sun exposure and Vitamin D”, “dietary Vitamin D”, “Vitamin D supplementation”, “bioavailability of Vitamin D in children”, “risk factors for dental caries”, and “preventive dentistry”.

Boolean operators were strategically applied to refine and connect MeSH search key terms effectively, as follows: (“Vitamin D” OR “serum 25(OH)D” OR “calcidiol”) AND (“dental caries” OR “tooth decay”) AND (“permanent teeth” OR “primary teeth”) AND (“children” OR “adolescents” OR “pediatric population”) AND (“nutritional status” OR “dental health” OR “oral hygiene” OR “caries prevention”) AND (“Vitamin D deficiency” OR “calcium metabolism” OR “phosphorus metabolism” OR “dietary Vitamin D” OR “Vitamin D supplementation”).

Following the Preferred Reporting Items for Systematic Reviews and Meta-Analyses (PRISMA) guidelines [19], this systematic review protocol was developed to ensure a structured, transparent, and reproducible methodology. This review was registered with the Open Science Framework (OSF) database, with the registration code osf.io/frhvc.

### 2.2. Eligibility Criteria and Definitions

The eligibility criteria for this systematic review were developed to select studies that provide insights into the correlation between serum Vitamin D levels and dental caries in the permanent teeth of children and adolescents aged 12 years and older, according to the existing literature regarding permanent dentition [18]. The inclusion criteria for this review were as follows: First, the study population must specifically include individuals in the age group of 12 to 19 years, focusing on those with permanent dentition. Second, the research must directly investigate the relationship between serum Vitamin D levels and the occurrence of dental caries in permanent teeth, encompassing studies that evaluate the impact of Vitamin D deficiency, the role of Vitamin D in dental health, and the effects of Vitamin D supplementation on the prevention of dental caries. Third, a variety of study designs were considered, including randomized controlled trials, observational studies, cohort studies, case–control studies, cross-sectional studies and longitudinal studies. Fourth, only studies employing validated measures or well-defined parameters for assessing this correlation were included, requiring detailed biochemical evaluations of Vitamin D and clinical assessments of dental caries. Finally, this review limited its scope to peer-reviewed articles published in English.

Conversely, this review excluded non-human studies, such as in vitro or animal research, to concentrate exclusively on human-related outcomes. Studies that do not specifically target the adolescent population with permanent dentition or that fail to differentiate the effects on permanent teeth depending on the 12–19 age range were also excluded. Additionally, research lacking specific, quantifiable outcomes related to serum Vitamin D levels and dental caries, or missing sufficient detail for a comprehensive evaluation, were excluded. Lastly, grey literature, including non-peer-reviewed articles, preprints, conference proceedings, general reviews, commentaries, and editorials, was also excluded. 

### 2.3. Data Collection Process

**Figure 1:**
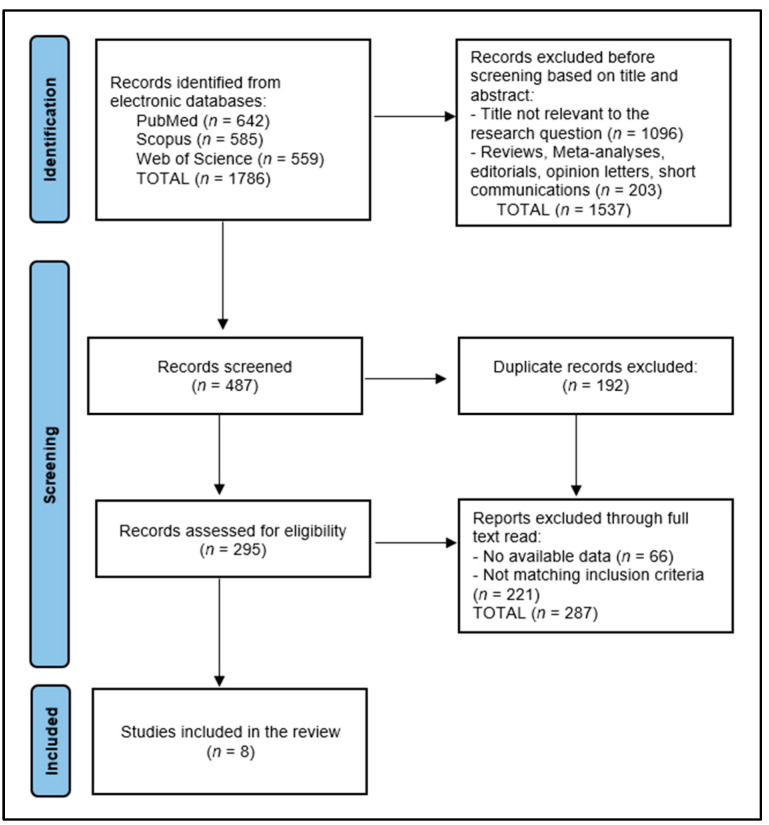
PRISMA flow diagram.

This systematic review began by eliminating 192 duplicate records. Subsequently, two independent reviewers conducted a detailed review of 295 abstracts to determine each study’s alignment with the established inclusion and exclusion criteria. Out of these, 287 records were discarded due to a lack of data (66 instances) or because they did not meet specific criteria concerning permanent dentition, patient age and Vitamin D levels in the serum. Any disagreements among the reviewers were settled through discussion or by consulting a third reviewer when needed to reach an agreement. The initial search of the database produced 1786 articles, ultimately narrowing down to 8 studies that were relevant and included in the final analysis, as shown in Figure 1.

### 2.4. Risk of Bias and Quality Assessment

In evaluating the quality of studies and identifying potential biases within them, our review adopted a mixed-method approach that combined qualitative and quantitative assessments. Initially, we assessed the quality of observational studies using the Newcastle–Ottawa Scale [20], which evaluates three key aspects: the selection of study groups, their comparability and the determination of exposures or outcomes for case–control or cohort studies, respectively. Studies were rated in these categories and received a cumulative score that categorized their quality as low, medium or high. To maintain the objectivity and reliability of our assessments, each study was independently reviewed by two researchers. Any differences in the assessment outcomes were addressed through discussions or, if required, by involving a third researcher.

## 3. Results

### 3.1. Study Characteristics

This systematic review analyzed a total of eight studies [21,22,23,24,25,26,27,28], detailed in Table 1. These studies were conducted across a variety of countries, including the United States [21,24], Portugal [22], the United Kingdom [23], Qatar [25], South Korea [26], China [27] and Korea [28], spanning the years 2013 to 2023. All identified studies employed a cross-sectional design, offering a snapshot of the relationship between serum Vitamin D levels and caries in the permanent teeth of children and adolescents at a single point in time.

The geographical diversity of these studies provides a broad perspective on the issue, allowing for an analysis that considers different dietary habits, sun exposure rates, and healthcare systems, which can influence Vitamin D levels and dental health outcomes. The timeframe of the studies, from 2013 to 2023, suggests a growing interest in this area of research, reflecting an increased recognition of the potential role of Vitamin D in dental health among the pediatric population.

The quality assessment of these studies varied, with most studies rated as medium quality and two studies [23,24] from the United Kingdom and the United States achieving a high-quality rating. This variation in study quality underscores the importance of critical appraisal in synthesizing evidence from existing research. 

### 3.2. Population Characteristics

This systematic review’s exploration of population characteristics across eight studies, as outlined in Table 2, encompasses an aggregate of 11,672 participants, providing a rich dataset for examining the relationship between serum Vitamin D levels and caries in permanent teeth among adolescents. The studies span a broad geographical range, from the United States [21,23] to Korea [26,28], each contributing unique insights into the demographic and environmental factors influencing Vitamin D synthesis and dental health outcomes.

The age ranges within these studies were carefully chosen to capture adolescents at crucial stages of dental development, with a notable focus on those with permanent dentition. Akinkugbe et al. [21] targeted a broad age range from 12 to 19 years, capturing a wide spectrum of adolescence. Gender distribution across the studies was relatively balanced, with a slight male predominance in some studies, such as Choi et al. [26], which reported 52.9% male participants. Study groups were often defined based on Vitamin D levels, with distinctions made between those with sufficient and insufficient levels. Carvalho Silva et al. [22], for example, distinguished participants based on 25(OH)D levels, identifying a significant portion (76.1%) with levels indicative of insufficiency or deficiency, highlighting the potential public health concern of Vitamin D inadequacy among adolescents. 

The studies’ geographical settings, ranging from the northern latitudes of Minnesota, USA [21], to the near-equatorial region of Qatar [25], provide a diverse context for examining the relationship between sunlight exposure, Vitamin D synthesis and dental health. Furthermore, the inclusion of risk ratios in some studies, such as the one reported by Al-Darwish et al. [25], indicating a higher caries risk among females (1.15 times higher than males), suggests the need for further investigation into gender-specific risk factors and protective mechanisms. 

### 3.3. Influencing Factors

The exploration of influencing factors on dental caries, as detailed in Table 3, across the included studies highlights a diverse array of lifestyle and environmental variables that could potentially impact the prevalence and severity of dental caries among adolescents. In the study by Akinkugbe et al. [21], a significant portion of the population reported high daily sugar consumption, with 54.5% consuming ≥120 g of sugar daily. This high intake is further compounded by the finding that 21.6% of the study population had serum cotinine levels above 3 ng/L, indicating exposure to tobacco smoke. Additionally, the study population’s racial composition, with 33.1% identifying as Black, provides a demographic context that may intersect with both environmental and genetic factors influencing caries risk.

Carvalho Silva et al. [22] reported that regular dental hygiene practices and dental visits were associated with a lower risk of caries, evidenced by a significant odds ratio of 2.57. The study also found that the consumption of cariogenic foods and drinks was linked to an increased risk of advanced caries and dental caries, with odds ratios of 1.20 for both. Notably, gastrointestinal disorders were significantly associated with advanced caries, with an odds ratio of 5.01, suggesting that systemic health conditions could play a critical role in dental health outcomes.

Herzog et al. [23] and Rigo et al. [24] both emphasized the importance of regular brushing, with the majority of their study populations brushing at least twice a day. Herzog et al. further reported that a substantial 82.2% of participants had visited a dentist within the past year, indicating a high level of engagement with dental care services. However, only a small fraction (5.7%) reported consuming more than 10% of their total daily food intake as sugar, which contrasts with the dietary habits observed in Akinkugbe et al. [21].

Al-Darwish et al. [25] highlighted a lower frequency of brushing (at least twice a day by only 35.3% of participants) and dental visits (18.7% in the past year), which might contribute to the observed dental health challenges within their study population. Moreover, a significant finding of calcium deficiency in 48.4% of their participants underscores the potential nutritional deficiencies impacting dental health beyond Vitamin D.

Choi et al. [26] and Kim et al. [28] provided insights into brushing frequency, with a notable portion of their populations adhering to the practice of brushing three times a day. This level of oral hygiene practice, however, did not translate into detailed reports on dental visits or the consumption of cariogenic foods and drinks. Pu et al. [27] did not report on these influencing factors, yet the racial composition of their study population, with 13.6% identifying as Black, adds another layer to the demographic analysis of dental caries risk factors. Overall, patients with dark skin colors represented a proportion ranging from 9.8% in the study by Al-Darwish et al. [25] to 33.1% in Akinkube et al. study [21].

### 3.4. Vitamin D and Dental Assessment

In the study by Akinkugbe et al. [21], the prevalence of Vitamin D deficiency and insufficiency was reported at 17.3% and 19.0%, respectively. The odds ratios (ORs) calculated for caries incidence in the context of Vitamin D insufficiency (OR = 1.02) and deficiency (OR = 1.23) suggest a slightly increased risk for caries among the deficient group. However, the study concludes that there is no significant association between serum Vitamin D levels and caries in permanent teeth, indicating that other factors may play a more dominant role in caries development within this population.

Conversely, Carvalho Silva et al. [22] found a strong association between Vitamin D insufficiency/deficiency (with 65.1% of the study population falling into these categories) and the occurrence of advanced caries, as evidenced by an OR of 2.27. Herzog et al. [23] presented a particularly interesting outcome, with an OR of 0.59 indicating a protective role against caries for individuals with Vitamin D levels above 50nmol/L. Rigo et al. [24], with a deficiency rate of 3.0% and an insufficiency rate of 23.9%, calculated an OR of 1.30 for caries, yet concluded that there was no significant association between serum Vitamin D and caries in permanent teeth. 

Al-Darwish et al. [25] observed a significantly higher risk of caries in the Vitamin D-deficient population, with an OR of 1.13 for caries. Choi et al. [26] reported a notably higher OR of 2.57 for caries in the context of Vitamin D insufficiency, indicating a significant association between lower Vitamin D levels and increased caries risk in the permanent dentition of their study population.

**Figure 2:**
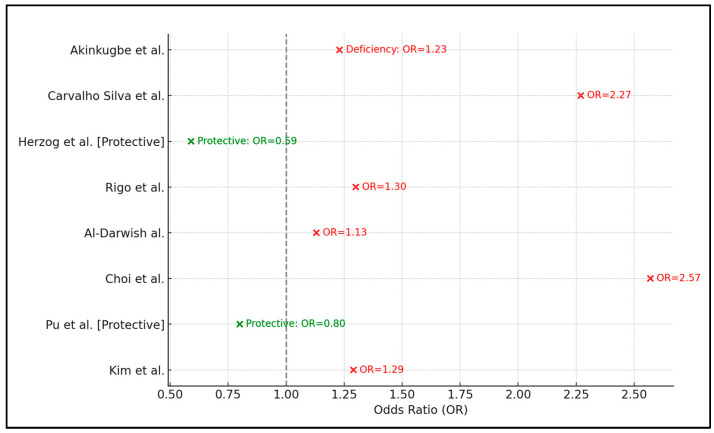
Protective and harmful effects of Vitamin D on caries development in permanent teeth of teenagers and adolescents [21,22,23,24,25,26,27,28].

Lastly, Pu et al. [27] presented evidence of a protective role of Vitamin D, with an OR of 0.80 for caries among individuals with levels ≥ 50 nmol/L, reinforcing the beneficial impact of sufficient Vitamin D levels on dental caries risk. Kim et al. [28], with 61.7% of the population being Vitamin D-insufficient, found a significant association between Vitamin D insufficiency and caries risk, with an OR of 1.29, as presented in Table 4 and Figure 2.

## 4. Discussion

### 4.1. Summary of Evidence

This systematic review unveils a nuanced landscape where the serum levels of Vitamin D among children and adolescents show a variable association with the incidence of caries in permanent teeth. Studies such as Carvalho Silva et al. [22] and Choi et al. [26] present compelling evidence that Vitamin D insufficiency and deficiency significantly elevate the risk of developing advanced dental caries. These findings underscore the critical role of adequate Vitamin D levels in maintaining oral health, potentially through its influence on the mineralization process of dental enamel and modulation of the immune response, which could offer protective mechanisms against the bacterial etiology of caries.

Conversely, the research outcomes from studies like Akinkugbe et al. [21] and Rigo et al. [24] suggest a more complex scenario where the direct correlation between Vitamin D levels and caries prevalence is not as clear-cut. In these instances, the observed slight increase in caries risk associated with Vitamin D deficiency does not reach statistical significance, indicating that factors beyond Vitamin D status may exert a more substantial influence on caries development. This divergence in findings across the reviewed studies emphasizes the multifactorial nature of dental caries, where dietary habits, oral hygiene practices, genetic predispositions and environmental factors converge to affect oral health outcomes. Moreover, these findings can be influenced by independent factors such as sun exposure [29], skin color [30], season of blood draw [31] and the geographical position of the study region [32].

Notably, Herzog et al. [23] and Pu et al. [27] offer an interesting counter-narrative by highlighting the protective role of sufficient Vitamin D levels against dental caries. Their results, showing a decreased odds ratio for caries in individuals with higher serum Vitamin D levels, align with the biological understanding of Vitamin D’s role in enhancing enamel calcification and anti-inflammatory responses. This protective association invites further investigation into Vitamin D as a modifiable risk factor for dental caries, suggesting that interventions aimed at improving Vitamin D status in youth could form a component of comprehensive caries-prevention strategies.

Other studies, such as the ones by Borsting et al. [33] and Li et al. [6], delved into the complex relationship between Vitamin D levels and oral health outcomes in children, yet they approached the topic from distinct perspectives and methodologies, including mixed dentition and younger patients than those included in our systematic review. Borsting et al. focused on a specific population of 7–9-year-old Norwegian children, employing Liquid Chromatography with Tandem Mass Spectrometry (LC-MS/MS) for precise Vitamin D measurement, and explored both the prevalence and the number of teeth affected by dental caries or MIH. Although this approach is ideal for a longitudinal study, the age range does not cover permanent dentition, which was the main focus of our study. Borsting’s study [29] identified no significant association between caries and insufficient Vitamin D serum levels. On the other hand, Li et al. [30] conducted a broader analysis through a meta-analysis of 13 studies, concluding that children with Vitamin D deficiency had a 22% higher risk of dental caries.

Another meta-analysis by Mahmood et al. [34] and the observational study by Chhonkar’s [35] both underline the significant association between Vitamin D deficiency and the risk of dental caries in children, although through different research lenses. Mahmood et al. analyzed 13 cross-sectional studies, finding that low serum Vitamin D levels increased the likelihood of dental caries (OR: 1.41), with each 10 nmol/L increase in serum Vitamin D reducing caries risk by 3% (OR: 0.97), although with no distinction between permanent and decidual dentition. In contrast, Chhonkar’s study focused on the decidual dentition of children aged 3 to 6 years, demonstrating a stark difference in mean serum Vitamin D levels between children with severe early childhood caries (12.19 ng/mL) and those without caries (20.11 ng/mL), with a statistically significant inverse correlation between Vitamin D levels and caries severity. These findings collectively highlight Vitamin D’s role in oral health, suggesting that maintaining adequate levels could significantly mitigate caries risk in children, with Mahmood et al. providing a broader epidemiological perspective and Chhonkar offering concrete clinical evidence within a specific demographic.

Ji et al. [36] and Sobiech et al. [37] delve into the association between Vitamin D levels and severe early childhood caries (S-ECC), yielding insights that underscore Vitamin D’s potential role in caries prevention. Ji et al. conducted a systematic review and meta-analysis, identifying significantly lower 25(OH)D levels in children with ECC compared to those who were caries-free, with a notable weighted mean difference of −13.96, indicating a strong correlation particularly with S-ECC (mean difference of −18.64). Their analysis suggested an optimal Vitamin D level ≥ 75 nmol/L for ECC prevention. Sobiech et al., [37] through a detailed survey and clinical examination, found a 44.8% occurrence of S-ECC in their study population, with Vitamin D supplementation and toothbrushing significantly reducing the odds of S-ECC (OR = 0.49 and OR = 0.46, respectively). While Ji et al. [36] provide a broad, quantitative analysis emphasizing the systemic relationship between Vitamin D and ECC across different regions, Sobiech et al. offer a practical perspective, linking Vitamin D supplementation directly with reduced S-ECC risk alongside other oral health behaviors. 

The decision to exclude younger patients from our systematic review was grounded in the specific aim to explore the relationship between serum Vitamin D levels and dental caries in the context of permanent dentition, which primarily emerges and stabilizes in individuals aged 12 years and older [19]. This age criterion allows for a focused examination of the adolescent population, where both the presence of permanent teeth is assured and the dietary and hormonal changes significant to Vitamin D metabolism and dental health are most pertinent. By distinguishing between deciduous and permanent teeth, this review targets a demographic where Vitamin D’s role in dental caries can be assessed more accurately, avoiding confounding factors associated with the deciduous dentition phase.

This systematic review’s insights suggest that incorporating Vitamin D status evaluation into adolescent dental care could enhance caries prevention efforts, advocating for the potential inclusion of Vitamin D supplementation in clinical practices and public health promotion efforts. As future research endeavors to establish a clearer causative link between Vitamin D levels and dental caries risk, clinicians are encouraged to consider Vitamin D’s role within a multifaceted approach to oral health. Exploring optimal Vitamin D thresholds for caries prevention and its interaction with dietary, hygienic and genetic factors is essential for reducing dental caries incidence in adolescents, thereby setting a direction for both future clinical practice and research.

### 4.2. Limitations

One notable limitation of this systematic review is the existence of only cross-sectional studies, which, while valuable for identifying associations at a specific point in time, do not enable the establishment of causality between serum Vitamin D levels and the incidence of dental caries in children and adolescents. This design limitation inherently restricts the ability to discern whether low Vitamin D levels precede caries development or result from dietary and lifestyle factors associated with poor oral health. Another limitation of this study was the decision to focus solely on three specific databases (PubMed, Scopus, Embase) and exclude others, particularly LILACS, which could have offered valuable insights. Additionally, by restricting the review to English-language articles, it may have missed important findings published in other languages, potentially introducing bias into the results and conclusions. Additionally, the heterogeneity in the methodologies and the quality of the included studies, with most rated as medium quality, may affect the generalizability of the findings. Variations in the definition of Vitamin D insufficiency and deficiency, assessment methods for dental caries, and the demographic and geographic diversity of the study populations further complicate direct comparisons and meta-analytic synthesis of the results. 

## 5. Conclusions

In conclusion, this systematic review comprises existing evidence that suggests a significant protective role of Vitamin D in caries development in children and adolescents with permanent dentition, when serum levels are above 20 ng/mL. Moreover, a significant risk association between Vitamin D insufficiency and deficiency and dental caries was observed, supporting the inclusion of Vitamin D assessments in the broader context of caries evaluation and management. Given the limitations inherent in the reviewed studies, there is a clear imperative for longitudinal research to better understand the causative mechanisms at play, alongside an exploration of how Vitamin D supplementation could be optimally integrated into public health strategies for caries prevention in the pediatric population, along with other prevention measures.

## Figures and Tables

**Table 1:** Study characteristics.

Study and Author	Country	Study Year	Study Design	Study Quality
1 Akinkugbe et al. [21]	United States	2018	CS	Medium quality
2 Carvalho Silva et al. [22]	Portugal	2021	CS	Medium quality
3 Herzog et al. [23]	United Kingdom	2022	CS	High quality
4 Rigo et al. [24]	United States	2023	CS	High quality
5 Al-Darwish et al. [25]	Qatar	2013	CS	Medium quality
6 Choi et al. [26]	South Korea	2018	CS	Medium quality
7 Pu et al. [27]	China	2023	CS	Medium quality
8 Kim et al. [28]	Korea	2018	CS	Medium quality

CS—cross-sectional.

**Table 2:** Population characteristics.

Study Number	Sample Size	Age Range	Gender Distribution	Study Groups (Vitamin D Levels)	Region (Latitude Range)
1 Akinkugbe et al. [21]	2579	12–14 years: 961 (36.8%) 15–19 years: 1618 (63.2%)	Female: 1261 (47.9%) Male: 1318 (52.1%)	Caries: 1357 No caries: 858	Minnesota—USA (43° N to 49° N)
2 Carvalho Silva et al. [22]	335 (67 with permanent dentition)	NR	Female: 167 (49.9%) Male: 168 (50.1%)	25(OH)D < 30 ng/mL: 51 (76.1%) 25(OH)D ≥ 30 ng/mL: 16 (23.9%)	Porto—Portugal (40° N to 42° N)
3 Herzog et al. [23]	3024	12–18 years	Female: 618 (48.5%) Male: 624 (51.5%)	Permanent teeth vs. primary teeth	District of Columbia—USA (38° N to 40° N)
4 Rigo et al. [24]	1242	12–19 years	Female: 618 (48.5%) Male: 624 (51.5%)	Permanent teeth vs. primary teeth	NR
5 Al-Darwish et al. [25]	492	13–16 years	NR (RR = 1.15 * higher caries risk for females)	NR	Qatar (24° N to 26° N)
6 Choi et al. [26]	1678	13–18 years	Female: 792 (47.1%) Male: 886 (52.9%)	Boys vs. girls	Korea (35° N to 40° N)
7 Pu et al. [27]	2748	15–19 years	Female: 48.7% Male: 51.3%	Caries: 1038 No caries: 1710	NR
8 Kim et al. [28]	574	12 years	Female: 46.6% Male: 53.4%	NR	Korea (35° N to 40° N)

NR—Not Reported; Vitamin D (25(OH)D) hypovitaminosis (insufficiency) is considered to be levels below 20 ng/mL or 50 nmol/L; Vitamin D deficiency is considered to be levels below 10 ng/mL or 25–30 nmol/L, according to Institute of Medicine threshold; RR = risk ratio; * - Statistically significant.

**Table 3:** Influencing factors.

Study Number	Brushing	Hygiene/Dental Visits	Food	Associated Factors	Skin Color
1 Akinkugbe et al. [21]	NR	NR	Daily sugar ≥ 120 g: 1349 (54.5%)	Serum cotinine > 3 ng/L: 452 (21.6%)	Black 33.1%
2 Carvalho Silva et al. [22]	NR	OR = 2.57 * (lower risk)	Cariogenic foods OR = 1.20 * for advanced caries Cariogenic drinks OR = 1.20 * for dental caries	Gastrointestinal disorders OR = 5.01 * for advanced caries	NR
3 Herzog et al. [23]	≥2 times/day: 66.3%	Dental visits (past year): 82.2%	More than 10% sugar intake (of total food consumption/day): 5.7%	NR	Black 14.2%
4 Rigo et al. [24]	≥2 times/day: 65.8%	NR	NR	NR	Black: 13.1%
5 Al-Darwish et al. [25]	≥2 times/day: 35.3%	Dental visits (past year): 18.7%	xxx	Calcium deficiency: 48.4%	Black: 9.8%
6 Choi et al. [26]	≥3 times/day: 36.4%	Dental visits (past year): 51.7%	NR	NR	NR
7 Pu et al. [27]	NR	NR	NR	NR	Black: 13.6%
8 Kim et al. [28]	≥3 times/day: 29.1%	NR	NR	NR	NR

NR—Not Reported; OR—odds ratio; *—statistically significant values.

**Table 4:** Vitamin D and dental assessment.

Risk Factors	Vitamin D Assessment	Caries	Risk Analysis (OR/RR)	Interpretation
1 Akinkugbe et al. [21]	Deficient: 17.3% Insufficient: 19.0%	Untreated decay on at least one tooth: 386 (14.9%) Dental restoration: 1130 (43.8%)	Insufficiency: OR = 1.02 for caries Deficiency: OR = 1.23 for caries	No significant association between serum Vitamin D and caries in permanent teeth.
2 Carvalho Silva et al. [22]	Insufficient and deficient: 65.1%	Dental caries: 23.9% Advanced caries: 20.0%	OR = 2.27 * for advanced caries	Advanced caries in permanent teeth was significantly associated with Vitamin D levels < 30 ng/mL.
3 Herzog et al. [23]	Deficient: 26.9% Insufficient: 74.9%	Dental caries: 53.6% Number of filled teeth: 3.4 (mean) ≥1 tooth sealed: 49.5%	OR = 0.59 * for caries	Significant protective role of Vitamin D above 50 nmol/L against caries.
4 Rigo et al. [24]	Deficient: 3.0% Insufficient: 23.9%	Dental caries: 21.7%	OR = 1.30 for caries	No significant association between serum Vitamin D and caries in permanent teeth.
5 Al-Darwish et al. [25]	Insufficient: 47.4%	Dental caries: 59% in Vitamin D-deficient population vs. 41% in non-deficient population	OR = 1.13 * for caries	Significantly higher risk of caries in permanent dentition of Vitamin D-deficient population.
6 Choi et al. [26]	Deficient: 21.5% Insufficient: 69.4%	Dental caries: 44.3% in boys, 49.5% in girls	OR = 2.57 * for caries	Significantly higher risk of caries in permanent dentition of Vitamin D-insufficient population.
7 Pu et al. [27]	Average 65.39 nmol/L	Dental caries: 55.6%	OR = 0.80 * for caries when Vitamin D is ≥50 nmol/L	Significant protective role of Vitamin D above 50 nmol/L against caries.
8 Kim et al. [28]	Insufficient: 61.7%	Dental caries: 51.7%	OR = 1.29 *	Significantly higher risk of caries in permanent dentition of Vitamin D-insufficient population.

Vitamin D refers to 25(OH)D levels; Vitamin D insufficiency (<20 ng/mL or 50 nmol/L); Vitamin D deficiency (10 ng/mL or <30 nmol/L); *—statistically significant values; NR—Not Reported; OR—odds ratio; RR—risk ratio.

## Data Availability

Data are contained within the article.
